# Deep learning-based prediction of TERT mutation status from MRI for glioma molecular subtyping

**DOI:** 10.3389/fneur.2026.1749556

**Published:** 2026-01-29

**Authors:** Ting Zhu, Xuhao Dai, Xiaoqin Ge, Yuqing Hu, Jiangping Ren, Jiming Yang, Ruishuang Ma, Qingsong Tao

**Affiliations:** Department of Radiotherapy and Chemotherapy, The First Affiliated Hospital of Ningbo University, Ningbo, China

**Keywords:** deep learning, glioma, molecular prediction, precision neuro-oncology, telomerase reverse transcriptase

## Abstract

**Background:**

This study aimed to develop and validate a deep learning model based on preoperative MRI to non-invasively predict Telomerase Reverse Transcriptase (TERT) promoter mutation status in glioma patients.

**Methods:**

A retrospective cohort of 100 patients with histologically confirmed high-grade glioma was included. Regions of interest (VOIs) were manually annotated on contrast-enhanced T1-weighted MRI sequences by senior radiologists. Five deep learning models (RegNet, GhostNet, MobileNet, ResNeXt50, ShuffleNet) were trained and evaluated using accuracy, precision, recall, and F1-score. The dataset was split into training (80%) and internal validation (20%) sets.

**Results:**

RegNet achieved the highest performance with an accuracy of 0.7742, recall of 0.8704, precision of 0.7163, and F1-score of 0.7023. It demonstrated superior ability to capture imaging features associated with TERT mutations compared to other models. The area under the ROC curve (AUC) for RegNet was 0.7182, indicating moderate discriminative power.

**Conclusion:**

The RegNet model effectively predicts TERT promoter mutation status from routine MRI, offering a non-invasive tool for preoperative molecular subtyping of glioma. This approach may facilitate personalized treatment planning and address limitations of invasive tissue-based diagnostics. Further validation with multi-center data is warranted to enhance clinical applicability.

## 1 Introduction

Glioma is one of the most common primary malignant tumors of the central nervous system and is classified by the World Health Organization (WHO) into low-grade and high-grade gliomas (HGG) (1–3). Glioblastoma (GBM), the most aggressive subtype of HGG, is characterized by marked genetic heterogeneity and poor prognosis, with a median survival of approximately 14 months despite multimodal treatment (4, 5).

Molecular classification has become central to glioma diagnosis, prognostic stratification, and treatment decision-making. Key molecular alterations include mutations in isocitrate dehydrogenase (IDH), co-deletion of chromosomal arms 1p/19q, EGFR amplification, chromosome +7/−10 changes, and telomerase reverse transcriptase (TERT) promoter mutations (6). Among these, TERT promoter mutation is one of the most frequent genetic events in adult gliomas and plays a crucial role in tumor progression by activating telomerase and enabling unlimited cellular proliferation (7–9). In high-grade gliomas, the incidence of TERT promoter mutations reaches approximately 70%, making it a critical marker for molecular subtyping and prognostic assessment of GBM (10–12).

Currently, the determination of TERT promoter mutation status relies primarily on postoperative molecular pathological analysis of tumor tissue obtained through surgical resection or biopsy. Although accurate, this approach is invasive, time-consuming, and associated with surgical risks, particularly for tumors located in deep or eloquent brain regions. As a result, reliable preoperative, non-invasive prediction of TERT mutation status remains a significant unmet clinical need.

With advances in artificial intelligence, radiomics and deep learning techniques have shown considerable promise in extracting high-dimensional imaging features from routine magnetic resonance imaging (MRI) to infer underlying molecular characteristics of gliomas (13–18). Several studies have explored MRI-based radiomics or deep learning models for predicting TERT promoter mutations, demonstrating encouraging performance (16, 19–21). However, most existing models are limited by single-architecture designs, heterogeneous imaging inputs, or lack of direct comparison among state-of-the-art deep learning frameworks. Moreover, non-invasive prediction of TERT promoter mutations specifically in high-grade gliomas remains relatively underexplored.

**Figure 1:**
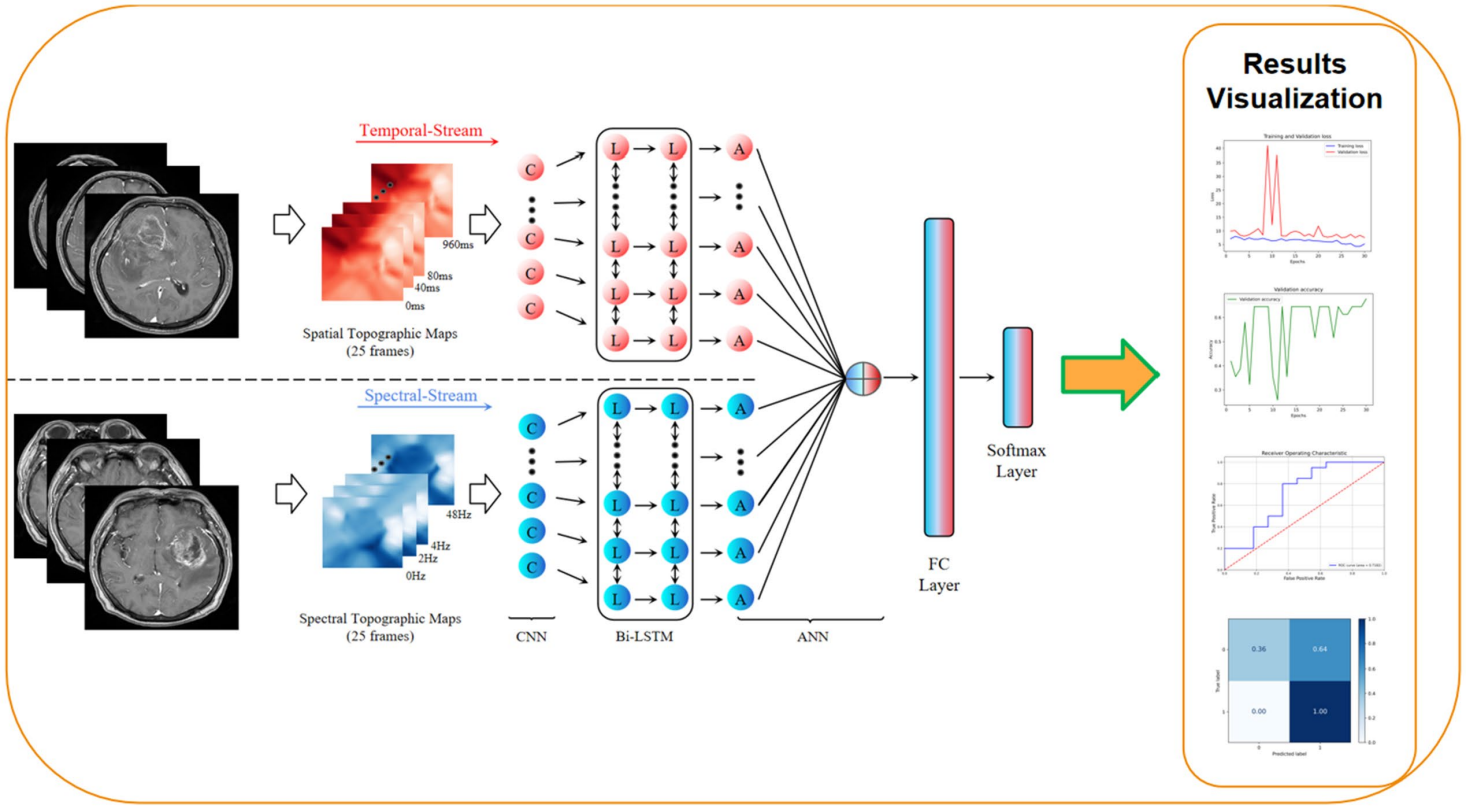
Workflow diagram of this study.

Therefore, the aim of this study is to develop and validate a deep learning model based on preoperative contrast-enhanced T1-weighted MRI (T1 + C) for predicting TERT promoter mutation status in patients with high-grade glioma. Using the RegNet architecture, we further conduct a systematic comparison with several advanced lightweight and mainstream convolutional neural networks, including GhostNet, MobileNet, ResNeXt50, and ShuffleNet. Our goal is to establish an efficient, accurate, and non-invasive imaging-based approach to assist molecular subtyping and personalized treatment planning in glioma management. The overall workflow of this study is illustrated in Figure 1.

## 2 Method

### 2.1 Study population

**Figure 2:**
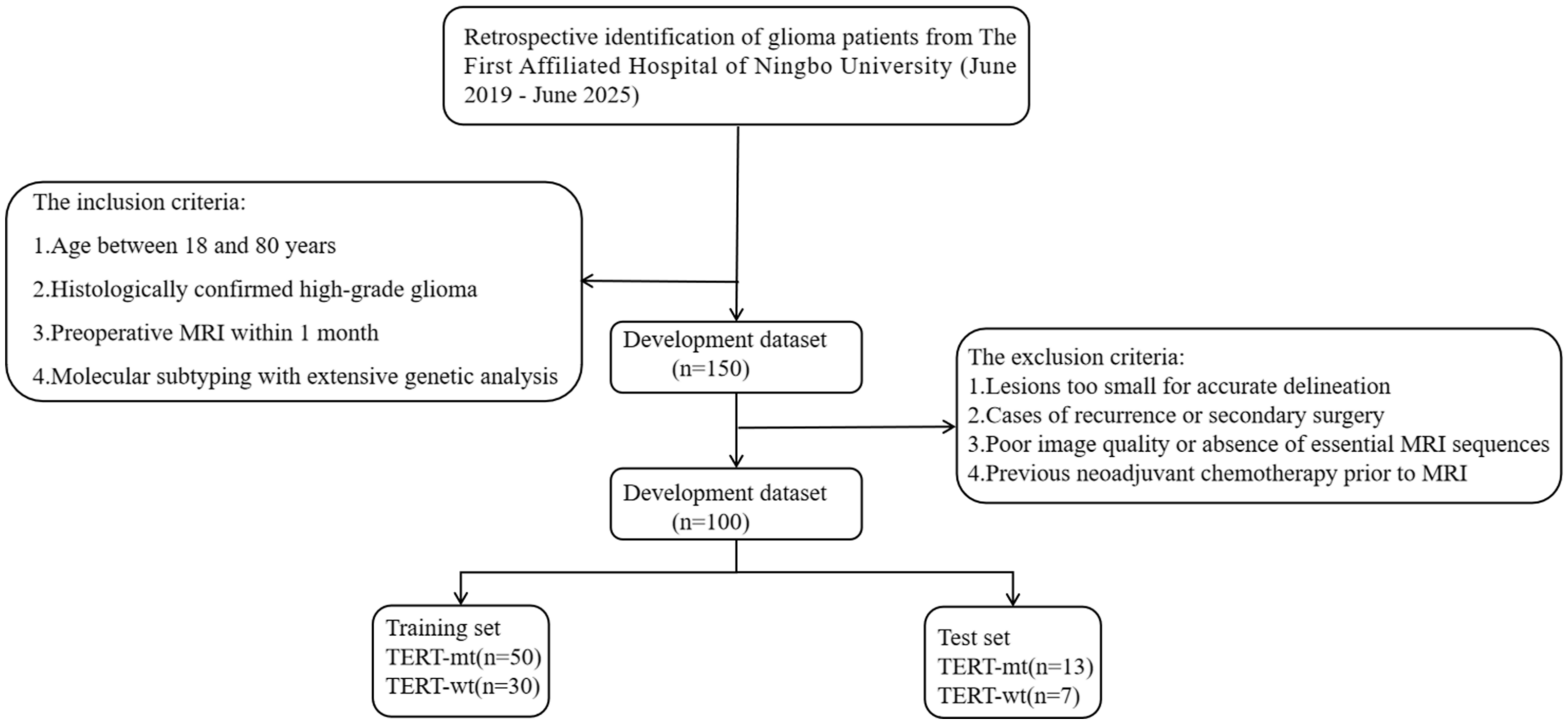
Patient enrollment flowchart.

This retrospective study initially identified 500 glioma patients who underwent surgical treatment at The First Affiliated Hospital of Ningbo University between June 2019 and June 2025. After applying predefined criteria—including age (18–80 years), histologically confirmed high-grade glioma, availability of preoperative MRI within 1 month before surgery, and complete molecular subtyping data—150 patients were preliminarily included. Further exclusions were applied to cases with lesions too small for reliable delineation (defined as a maximum axial diameter < 10 mm on contrast-enhanced T1-weighted MRI), recurrent tumors or secondary surgeries, poor image quality or missing key MRI sequences, and any prior neoadjuvant treatment, including chemotherapy or radiotherapy. After applying these criteria, a total of 100 patients were included in the final cohort. These 100 patients were randomly allocated into a training set (*n* = 80) and a test set (*n* = 20) in a 8:2 ratio. The study was approved by the hospital ethics committee (Approval No: 2025-145RS-01). The patient enrollment flowchart of this study is shown in Figure 2.

The molecular profiling of glioma includes determining the mutation status of the Telomerase Reverse Transcriptase (TERT) gene, a key genetic alteration associated with tumor progression and patient outcomes. This retrospective investigation classified post-operative glioma patients into two groups according to their TERT promoter status: a “TERT-mutant” group (TERT-mt) and a “TERT wild-type” group (TERT-wt). The study aimed to construct a predictive model for TERT gene mutations, which constitutes a binary classification task. The requirement for informed consent was waived for this study, which was approved by the hospital’s ethics committee.

### 2.2 Data preprocessing and lesion annotation

#### 2.2.1 Data preprocessing

This study implemented a standardized image preprocessing workflow to optimize MRI data for deep learning applications. Firstly noise reduction was performed using a non-local means (NLM) filter to minimize artifacts while maintaining anatomical integrity. Subsequently, edge enhancement was conducted through Laplacian filtering to accentuate morphological boundaries in lymph node regions. Following this, intensity normalization was applied to standardize voxel values across all scans to a consistent [0,1] range, thereby mitigating technical variations from different acquisition protocols. Additionally, spatial standardization was achieved through bicubic interpolation to resize images to 224 × 224 pixels, with careful ROI extraction to eliminate extraneous background information. ​​Finally, to enhance model generalizability, training incorporated comprehensive data augmentation strategies including random affine transformations (±15°rotation, 0.8–1.2 × scaling), horizontal flipping, and gamma correction, effectively expanding dataset diversity and improving robustness to anatomical and technical variations.

#### 2.2.2 Region of interest labeling

To precisely delineate glioma regions for TERT mutation status prediction, manual annotation of the volumetric region of interest (VOI) was performed on brain MRI scans using the contrast-enhanced T1-weighted (T1 + C) sequence. This process was carried out independently by two senior radiation oncologists, each with more than 10 years of experience in neuro-oncology, using 3D Slicer software (version 5.8.1). The clinicians employed multi-planar reconstruction (MPR) to visualize tumor morphology in axial, sagittal, and coronal views, enabling accurate spatial localization of the lesion. Delineation was restricted to areas of visible contrast enhancement on T1 + C images, carefully excluding adjacent normal brain parenchyma, peritumoral edema, necrotic regions, and any non-enhancing or distant suspicious foci. All annotated volumes were saved in NIfTI format. In cases of inter-observer variability, a consensus review was conducted to establish the final VOI, thereby ensuring both geometric consistency and anatomical validity for subsequent feature extraction.

### 2.3 Deep learning model construction

#### 2.3.1 RegNet model application

In this study, we employed the RegNet architecture as the core deep learning model for predicting TERT promoter mutation status in glioma based on MRI data. RegNet is a convolutional neural network (CNN) designed through systematic network architecture search and parameterized optimization, offering a balance between computational efficiency and feature representation capability. The model consists of stacked residual blocks, each containing convolutional layers, batch normalization, and residual connections to facilitate gradient flow during training (22). Using the RegNet-X-4.0GF variant, the network processes input images at a resolution of 224 × 224, enabling hierarchical extraction of discriminative features related to genetic alterations. The ReLU activation function and Adam optimizer were applied, with an initial learning rate of 1e-3 and a dynamic learning rate scheduling strategy to stabilize training. RegNet offers several advantages for glioma genetic phenotype prediction. Its modular and scalable design allows flexible adjustment of depth and width according to data volume and complexity, making it suitable for medical imaging tasks with limited samples. The residual connections and batch normalization layers enhance training stability and accelerate convergence, which is critical in clinical imaging analysis. Moreover, RegNet’s hierarchical feature extraction mechanism effectively captures both local texture details and global contextual information from MRI, which may reflect underlying molecular characteristics such as TERT mutation. By progressively aggregating multi-scale features, the model can identify subtle imaging patterns associated with genetic subtypes, improving prediction accuracy and robustness. These properties make RegNet well-suited for high-dimensional MRI-based genotype-prediction tasks in neuro-oncology.

#### 2.3.2 Other comparative models

In this study, we conducted a comparative analysis of several advanced deep learning architectures—GhostNet, MobileNet, ResNeXt50, and ShuffleNet—to evaluate their applicability in predicting TERT promoter mutation status in glioma based on MRI data. The following section details the core principles, comparative strengths, and potential limitations of each model in the context of this specific medical image analysis task.

GhostNet employs a novel Ghost Module, which first applies regular convolution to generate a set of intrinsic feature maps, followed by efficient, linear operations (cheap transformations) on each intrinsic map to produce additional “ghost” features. This design significantly reduces computational cost and model parameters while maintaining a rich feature representation. Its efficiency makes it particularly suitable for scenarios with limited computational resources. However, the feature representation capacity of the ghost features might be less expressive than those learned by full convolutions, which could be a limitation when extracting very subtle imaging biomarkers associated with TERT mutations from MRI. The MobileNet family, including its variants, is built upon depthwise separable convolution. This operation factorizes a standard convolution into a depthwise convolution (applying a single filter per input channel) followed by a pointwise convolution (1×1 convolution to combine channel outputs). This factorization leads to a massive reduction in computational complexity and model size. While highly efficient, its performance in complex tasks can be constrained by its lower parameter count and inherent limitations in modeling intricate feature interactions compared to denser models. ResNeXt50 is an evolution of the ResNet architecture that introduces cardinality as a new dimension. It adopts a split-transform-merge strategy using grouped convolutions within its residual blocks. This design increases the width of the network and enhances its feature representation power without a proportional increase in computational complexity. It strikes a balance between depth, width, and computational efficiency, making it a powerful backbone for capturing complex and hierarchical patterns in medical images, such as those potentially indicative of genetic mutations. ShuffleNet is designed for extreme efficiency on computational-constrained devices. Its core innovations are pointwise group convolutions and a channel shuffle​​ operation. Pointwise group convolutions reduce the cost of 1 × 1 convolutions, while the channel shuffle operation enables information flow across different groups, mitigating the side effects of grouped convolutions. This architecture achieves very high computational efficiency, but this can sometimes come at the cost of a slight drop in accuracy on challenging datasets where rich feature representation is critical.

### 2.4 Statistical analysis

To comprehensively evaluate the performance of our deep learning model in predicting TERT promoter mutation status in glioma from MRI data, we employed four widely recognized classification metrics: Accuracy, Precision, Recall, and F1-score. These metrics provide a multi-faceted assessment of the model’s diagnostic reliability, which is critical for clinical decision-making. Accuracy quantifies the overall proportion of correct predictions (both TERT mutant and wild-type) across all glioma cases. While it offers a general performance overview, its interpretation is made with consideration of potential class imbalance in the dataset. Precision measures the reliability of a positive prediction (i.e., TERT mutation). A high precision indicates that when the model predicts a TERT mutation, it is highly likely to be correct, thereby reducing the risk of false alarms that could lead to unnecessary patient anxiety or overtreatment. Recall (or Sensitivity) assesses the model’s ability to correctly identify all cases that truly harbor a TERT mutation. In a clinical context, high recall is paramount, as missing a true TERT mutation (a false negative) could have significant consequences for prognosis assessment and treatment planning. F1-score, as the harmonic mean of precision and recall, provides a single metric that balances the trade-off between these two concerns. It is particularly informative for evaluating performance on the positive class (TERT mutation) in potentially imbalanced datasets. The Equations 1–4 for these metrics are defined as follows:


$$\text{Precision}=\frac{\mathrm{TP}}{\mathrm{TP}+\mathrm{FP}}$$ (1)

$$\text{accuracy}=\frac{\mathrm{TP}+\mathrm{TN}}{\mathrm{TP}+\mathrm{TN}+\mathrm{FP}+\mathrm{FN}}$$ (2)

$$\begin{array}{c} \text{Recall}=\frac{\mathrm{TP}}{\mathrm{TP}+\mathrm{FN}} \end{array}$$ (3)

$$\begin{array}{c} F1=2x\frac{\text{Precision}\;x\;\text{Recall}}{\text{Precision}+\text{Recall}} \end{array}\;$$ (4)

### 2.5 Experimental setup

To ensure a fair and reproducible evaluation of deep learning models for predicting TERT promoter mutation status in glioma from MRI data, a standardized experimental protocol was implemented. The dataset was randomly split into 80% for training and 20% for testing. Model hyperparameters were optimized via a combination of grid and random search, and all models were trained using the Adam optimizer with an initial learning rate of 1 × 10–4, which was dynamically adjusted during training. A class weighting strategy was applied to the loss function to mitigate potential dataset imbalance. All experiments were run on a high-performance computing platform featuring an NVIDIA A100 GPU, utilizing the PyTorch 1.8.0 framework within a Linux environment with CUDA 11.2. This consistent setup ensures that performance comparisons are attributable to model architectures rather than experimental variations.

## 3 Results

### 3.1 Patients baseline clinical characteristics

Table 1 compares the baseline demographic and molecular characteristics between the training set (*n* = 80) and the internal test set (*n* = 20). Regarding age distribution, the mean age of patients in the training set was 59.81 ± 12.81 years, while in the test set, it was 59.20 ± 11.19 years. When categorizing patients by age 60, 46 patients (57.5%) in the training set and 11 patients (55%) in the test set were over 60 years old. The difference in age distribution between the two sets was not statistically significant (*p* > 0.05). In terms of sex distribution, the training set consisted of 37 males (46.25%) and 43 females (53.75%). The test set included 8 males (40%) and 12 females (60%). The difference in sex distribution was also not statistically significant (*p* > 0.05). For the TERT promoter mutation status, the mutant-type (TERT-mt) group comprised 50 patients (62.5%) in the training set and 13 patients (65%) in the test set. The wild-type (TERT-wt) group included 30 patients (37.5%) in the training set and 7 patients (35%) in the test set. The difference in the distribution of TERT status between the two sets was not statistically significant (*p* > 0.05). In conclusion, the baseline characteristics, including age, sex, and TERT promoter status, were well-balanced between the training and test sets, with no statistically significant differences observed (all *p*-values > 0.05). This suggests that the dataset split was appropriate for model training and validation.

**Table 1:** Patients baseline clinical characteristics statistics.

Characteristic	Train set (*n* = 80)		Test set (*n* = 20)		*P*-value
	NO.	%	NO.	%	
Age (mean ± SD)	59.81 ± 12.81		59.20 ± 11.19		
>60	46	57.5	11	55	<0.05
<60	34	42.5	9	45	
Sex					
Male	37	46.25	8	40	<0.05
Female	43	53.75	12	60	
Group					
TERT-mt	50	62.5	13	65	<0.05
TERT-wt	30	37.5	7	35	

### 3.2 Performance of RegNet in the test set

**Figure 3:**
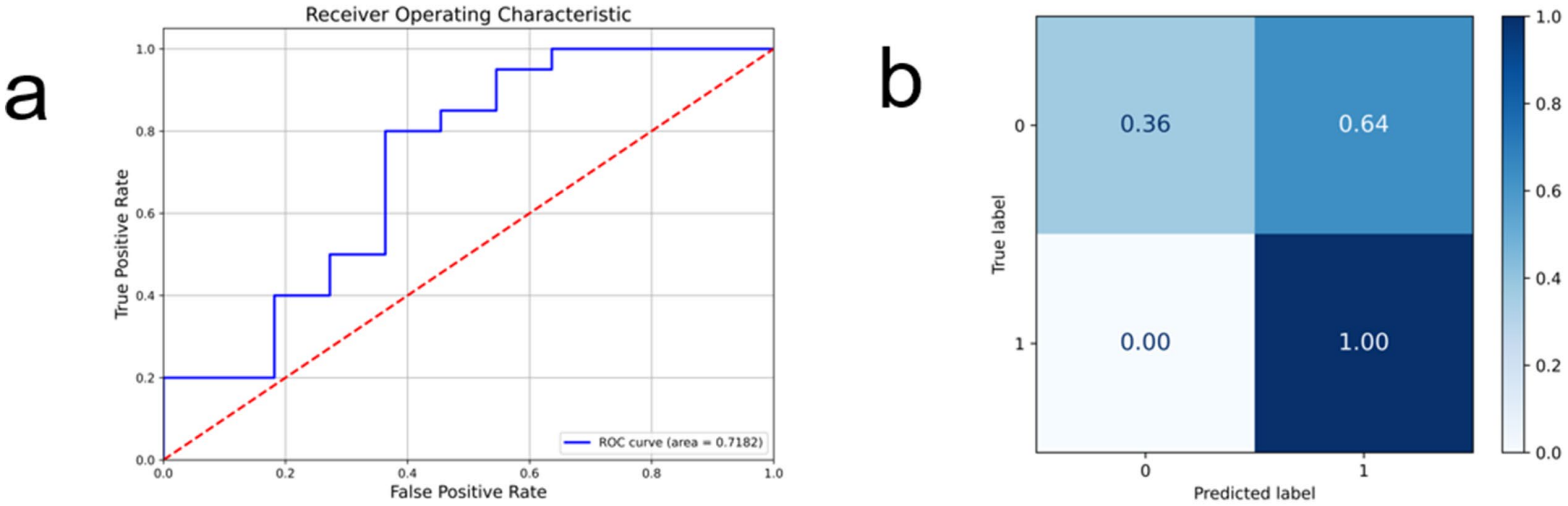
RegNet test results, **(a)** represents the ROC curve of the model and its AUC value, **(b)** reflects its confusion matrix.

In this study, the performance of the RegNet model for predicting TERT mutation status in glioma based on MRI is illustrated in the figure. Figure 3a presents the receiver operating characteristic (ROC) curve, with an area under the curve (AUC) of 0.7182, indicating a moderate discriminative ability of the model in distinguishing TERT mutant from wild-type cases. The curve exhibits a rapid initial rise, reflecting a high true positive rate at low false positive rates. Figure 3b displays the normalized confusion matrix expressed in percentages. Among the 20 test cases, the model correctly predicted 7 true negative samples (36%) and 20 true positive samples (100%), with no false negatives and 12 false positives (64%). The absence of false negatives suggests high sensitivity of the model in identifying TERT mutations, while the relatively high false positive rate may be attributed to challenging cases with ambiguous imaging features. These results demonstrate the potential of the proposed deep learning approach in non-invasively predicting TERT mutation status from routine MRI scans.

### 3.3 Comparing the performance of the models in the test set

**Figure 4:**
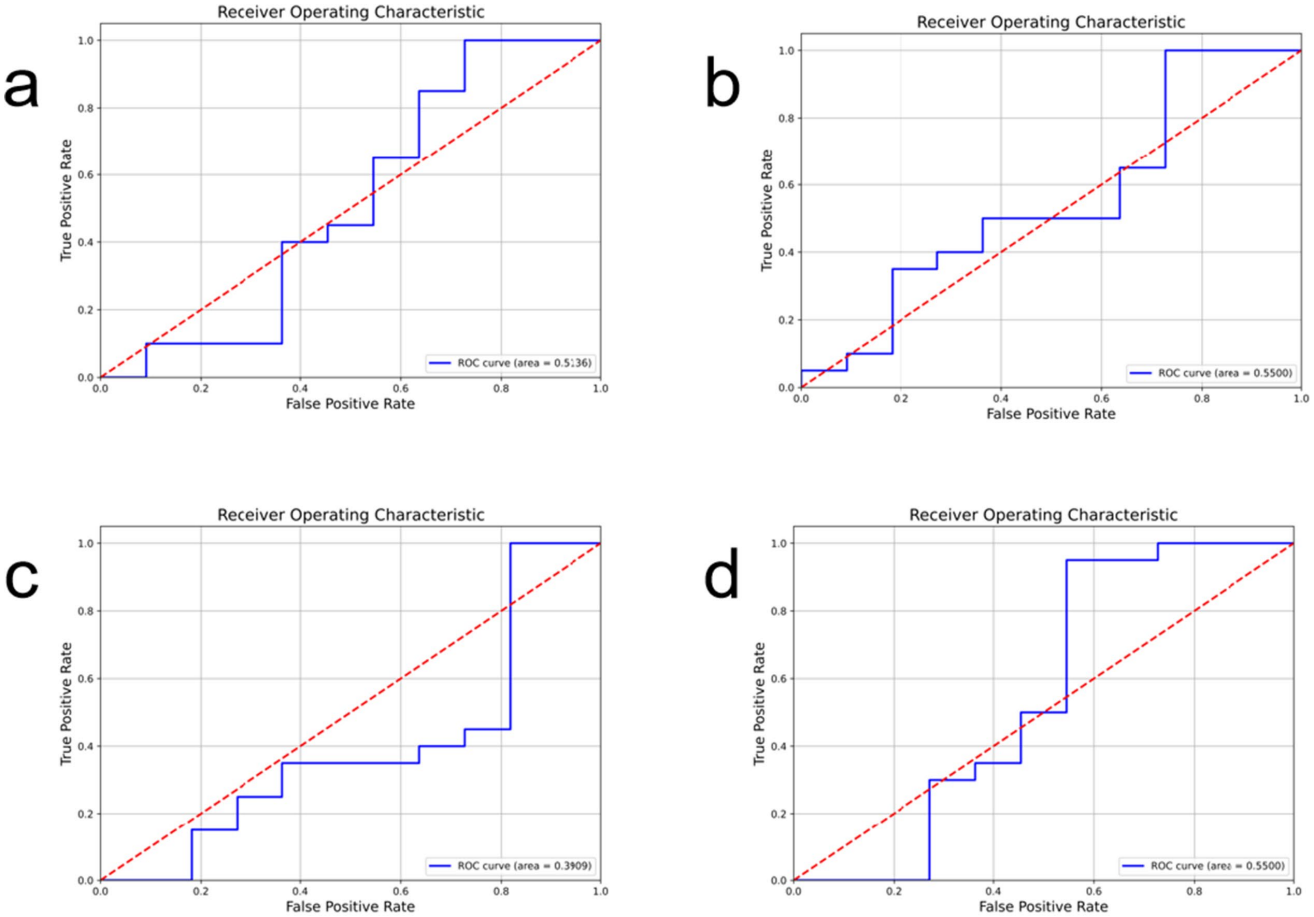
The ROC curves of the comparison models are shown, where **(a)** represents GhostNet, **(b)** represents MobileNet, **(c)** RegNet50, **(d)** ShuffleNet.

**Figure 5:**
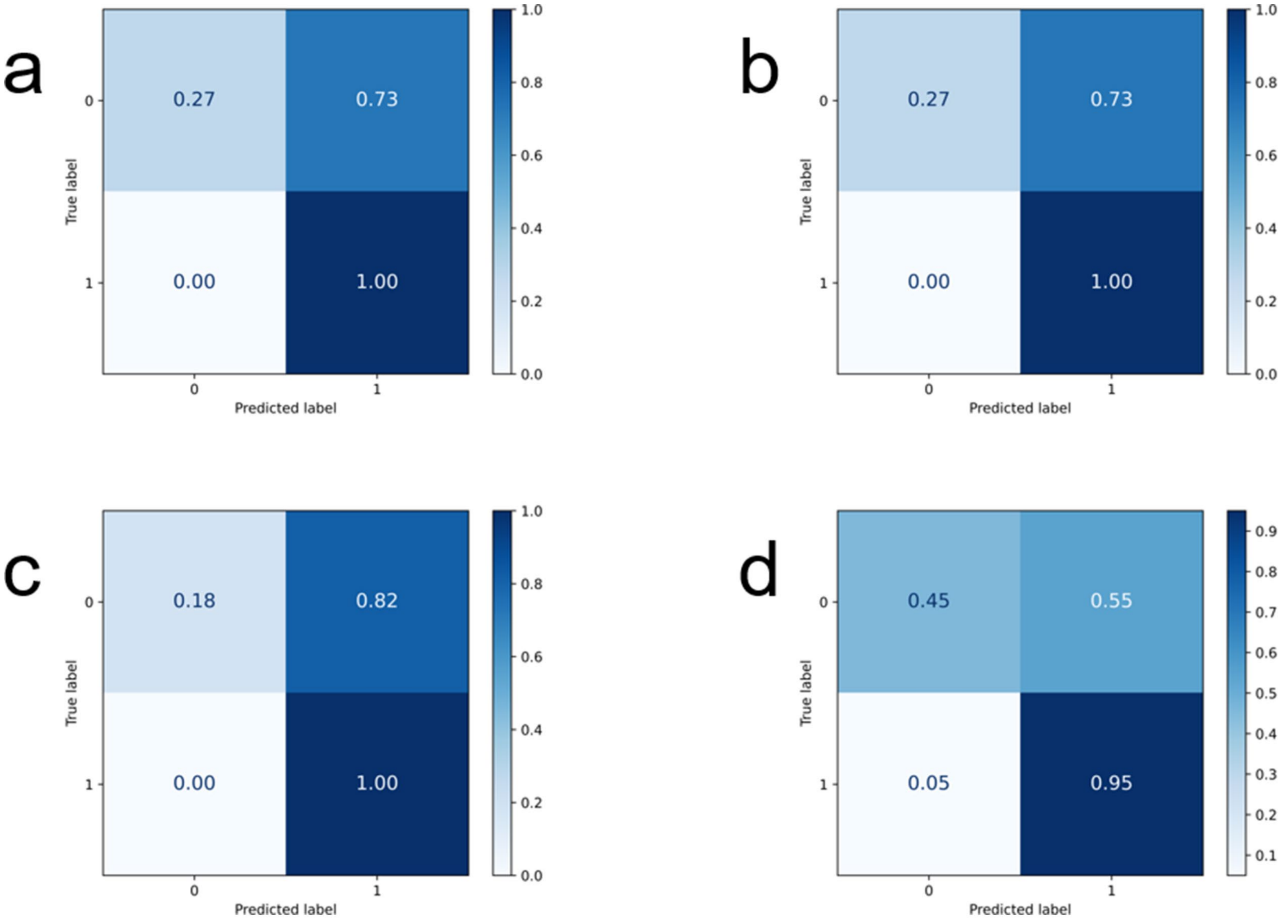
Confusion matrix display of each comparison model: **(a)** Represents GhostNet, **(b)** represents MobileNet, **(c)** RegNet50, **(d)** ShuffleNet.

Figures 4, 5 show the ROC curves and confusion matrices of the comparison models. Table 2 shows the evaluation indicators of all models. Among the comparison models used, GhostNet and MobileNet showed identical results across all metrics (accuracy: 0.7419, recall: 0.8571, F1-score: 0.6364, precision: 0.631), suggesting similar predictive capabilities for this specific task. Meanwhile, ResNeXt50 delivered competitive accuracy (0.7742) but had lower recall and F1-score compared to RegNet. The ShuffleNet architecture exhibited the lowest performance across all evaluated metrics. In conclusion, the comparative analysis clearly identifies RegNet as the optimal model for this application, striking the most effective balance between correctly identifying mutations (high recall) and ensuring prediction reliability (high precision), as reflected in its leading F1-score.

**Table 2:** Performance comparison of various models.

Model	acc	recall	f1-score	precision
GhostNet	0.7419	0.8571	0.6364	0.631
MobileNet	0.7419	0.8571	0.6364	0.631
ResNeXt50	0.7742	0.7967	0.6818	0.6922
ShuffleNet	0.7097	0.8448	0.5909	0.562
**RegNet**	**0.7742**	**0.8704**	**0.7023**	**0.7163**

Bold values indicate the best value in each column.

## 4 Discussion

Based on magnetic resonance imaging (MRI) data, this study developed and evaluated multiple deep learning models for the non-invasive prediction of TERT promoter mutation status, a critical molecular subtype in glioma. A comparative performance analysis of five network architectures—GhostNet, MobileNet, RegNet50, ShuffleNet, and RegNet—was conducted. The results demonstrated that the RegNet architecture achieved superior overall performance, with the highest accuracy (0.7742) and F1-score (0.7023) among the compared models. It also attained the top recall (0.8704) and precision (0.7163), indicating an optimal balance between correctly detecting mutation-positive cases and minimizing false-positive predictions. This outcome suggests that RegNet can effectively extract subtle imaging features from routine MRI scans, potentially capturing critical information related to morphological changes, tumor margins, and tissue density associated with TERT promoter mutations.

From an architectural perspective, RegNet employs a highly flexible and regularized design strategy, contributing to its computational efficiency across various hardware platforms. Its core innovation lies in the “design space design” paradigm. Unlike designing a single network instance, this approach systematically identifies generalizable, quantized linear rules governing the relationship between network depth and width in high-performing models, leading to a more interpretable and efficient structure. This modular and rule-based design reduces model redundancy and computational overhead while maintaining powerful representational capacity. Such optimization is particularly advantageous for medical image analysis, as it enhances the model’s ability to capture fine-grained details essential for identifying often subtle textural or morphological micro-variations indicative of TERT promoter mutations on MRI. For a binary classification task like predicting TERT mutation status, RegNet demonstrates excellent adaptability: its deep architecture enables the progressive extraction of hierarchical features from low-level to high-level, which is crucial for discerning minor differences in MRI data. Furthermore, the model’s parameter efficiency helps mitigate overfitting risks, a significant advantage when working with limited annotated medical imaging datasets, thereby enhancing the model’s generalizability.

Tang et al. developed conventional MRI–based radiomics models for predicting TERT promoter mutation in gliomas, reporting modest performance with an RFE–linear regression model (AUCs: 0.733/0.562/0.633 in training/validation/testing sets) (23). Zheng et al. proposed a transcriptome-based qualitative signature of 21 gene pairs to identify high-risk TERT promoter mutant low-grade gliomas, successfully stratifying patients into prognostically distinct groups with GBM-like features (24). Nakagaki et al. integrated whole-slide histopathology images and clinical data using deep learning to predict IDH1 mutation status, achieving improved performance with a MaxViT–LightGBM ensemble model (AUC = 0.852) (25). Notably, a systematic review by Kalaroopan and Lasocki highlighted that despite encouraging results, most MRI-based deep learning studies for glioma genotyping rely on small or public datasets and lack independent validation, reflecting the challenges of assembling large, high-quality imaging–genomic cohorts (26).

Although prior studies have demonstrated the feasibility of MRI-based radiogenomic prediction in gliomas, several methodological limitations remain. Many investigations rely on relatively simple machine learning models, resulting in suboptimal predictive performance. A substantial proportion are single-center studies without external validation, which limits generalizability. Moreover, most existing work—particularly those based on public datasets—has focused on the prediction of single molecular alterations, such as IDH1 mutation or MGMT promoter methylation. Public datasets often suffer from incomplete molecular annotations, missing genetic variables, and heterogeneity in testing platforms or diagnostic criteria. In contrast, privately curated datasets with comprehensive and standardized molecular profiling remain scarce, posing a significant challenge to the development and validation of robust, clinically translatable radiogenomic models. Furthermore, some studies rely on invasive tissue-based assays to obtain molecular information, which precludes truly non-invasive prediction and limits the clinical applicability of these approaches. Despite a prolonged enrollment period of 5 years, only 100 patients with high-grade glioma from our single center met the inclusion criteria of having both pre-treatment MRI data and complete molecular profiling. This reflects a realistic clinical challenge. Neurosurgical management of gliomas is technically demanding and requires advanced surgical and pathological infrastructure, limiting the ability of many institutions to systematically accumulate high-quality imaging–genomic datasets. In addition, molecular profiling involves sensitive genetic information, and data sharing is often constrained by ethical approval processes, patient privacy concerns, and regulatory barriers, which further complicate multi-center collaboration.

In conclusion, this study demonstrates the feasibility of using RegNet-based deep learning models to explore the non-invasive prediction of TERT promoter mutation status from standard MRI. While RegNet showed relatively better performance among the evaluated architectures, these findings require confirmation through external validation and larger multicenter studies before clinical translation. With future expansion of sample size and multi-center data integration, combining imaging-based prediction of IDH status, TERT promoter mutation, EGFR amplification, and chromosome 7/10 alterations may enable a more comprehensive non-invasive molecular stratification framework for glioblastoma (GBM). Moreover, integrating molecular prediction models with longitudinal post-treatment imaging holds promise for more clinically impactful applications, such as differentiating therapy-induced changes from true tumor recurrence, which represents an important direction for future research.

## 5 Conclusion

This study demonstrates the potential of deep learning models, particularly RegNet, to non-invasively predict TERT promoter mutations in glioma using standard MRI data. RegNet’s optimized architecture enabled robust feature extraction from tumor regions, achieving a balance between sensitivity (recall: 0.8704) and predictive reliability (precision: 0.7163). The model’s performance underscores the value of integrating computational methods with neuro-oncology to address challenges in molecular subtyping, such as surgical risks and diagnostic delays associated with tissue-based techniques. However, limitations including a small single-center sample and reliance on single-modality MRI highlight the need for future work involving multi-center cohorts and multi-parametric imaging (MRS, DTI). Prospective clinical trials are essential to validate the model’s utility in real-world settings and explore its integration into diagnostic workflows. Ultimately, this AI-driven approach could contribute to precision oncology by enabling preoperative genetic characterization, particularly for inoperable patients or cases where invasive procedures are contraindicated.

## Data Availability

The datasets presented in this article are not readily available because there are patient privacy-related concerns with sharing the data. Requests to access the datasets should be directed to contact the corresponding author at the email tqs3932@163.com.
